# Risk Assessment for Molds in the Vicinity of a Child Requiring Peritoneal Dialysis Living in a Rural Northern German Area

**DOI:** 10.3390/microorganisms9112292

**Published:** 2021-11-04

**Authors:** Andreas Erich Zautner, Hagen Frickmann, Andreas Podbielski

**Affiliations:** 1 Institute of Medical Microbiology and Hospital Hygiene, Medical Faculty, Otto-von-Guericke University Magdeburg, 39120 Magdeburg, Germany; 2 Department of Microbiology and Hospital Hygiene, Bundeswehr Hospital Hamburg, 20359 Hamburg, Germany; frickmann@bnitm.de; 3 Institute for Medical Microbiology, Virology and Hospital Hygiene, University Medicine Rostock, 18057 Rostock, Germany; andreas.podbielski@med.uni-rostock.de

**Keywords:** invasive mycoses, *Lichtheimia*, *Aspergillus*, Mucorales, environmental investigation, prevention

## Abstract

As well as severe immunosuppression, other predisposing factors may facilitate invasive mycosis caused by molds. Chronic kidney disease and the resulting peritoneal dialysis have been reported as factors putting patients at risk of fungal infections from environmental sources. We describe an environmental investigation undertaken to guide exposure prevention for a peritoneal dialysis patient with transient colonization of her nostrils by *Lichtheimia corymbifera* in a rural area of northern Germany. Systematic screening for airborne and surface-deposited molds enabled targeted recommendations to be made, although *Lichtheimia corymbifera* itself was not grown from the collected environmental samples. This communication is intended to illustrate how such an investigation can be performed on the basis of the environmental distribution of the molds and how preventive recommendations can be derived from the results.

## 1. Introduction

As has been described previously, children undergoing peritoneal dialysis are at risk of acquiring systemic mold infections [1]. Dialysis-associated systemic mycoses have been described for various molds, including *Aspergillus* spp., *Fusarium* spp., Mucorales and *Penicillium* spp. [2,3,4,5,6]. Here, we describe an environmental screening process prompted by the nasal colonization of a child undergoing peritoneal dialysis with *Lichtheimia corymbifera*, a member of the order Mucorales; this was conducted in order to reduce the child’s risk of exposure to environmental molds and the associated risk of progression to systemic infection.

Mucorales can cause severe invasive infections in immunocompromised patients [7,8,9], including severely mutilating rhino–orbital–cerebral lesions [10,11,12] as well as pulmonal, cutaneous, gastrointestinal, disseminated, and other manifestations [13]. Mucormycosis is globally distributed, occurring in temperate climates [14] and in sub-tropical or tropical settings [15,16,17]. The prognosis for survival is usually poor [7] and therapeutic options are limited to newer azoles such as posaconazole and isavuconazole as well as liposomal amphotericin B [7,18,19,20,21,22,23]. To date, however, neither the FDA (Food and Drug Administration) nor the EMA (European Medicines Agency) have granted approval for first-line posaconazole therapy. As well as immunosuppression [24,25], the availability of ionic iron has been reported to be critical for the onset of mucormycosis [26]. Even in apparently immunocompetent hosts, gastrointestinal mucormycosis [27] and other variants of Mucorales infections [28] have been described, although adaptive and innate immunity usually prevents severe infections in individuals who do not have predisposing factors [29]. Children can also be affected [30,31], again with severe immunosuppression as the underlying medical condition [32]. Inoculation of fungal spores via the skin due to traumatic injuries or burns is the typical route of infection, even in patients without immunosuppression. [14]. Severe hyperglycemia or ketoacidosis, as well as iron overload resulting from repeated blood transfusions and blood disorders, have also been recorded in association with mucormycosis in immunocompetent hosts [33,34]. Diabetes mellitus, in particular, has been considered as a major risk factor in various reviews [35,36], whereas in an Asian study, post-pulmonary tuberculosis and chronic kidney disease were reported as further predisposing factors [37]. Indeed, several cases of peritoneal-dialysis-associated mucormycosis have been described [6,38,39,40]. Most recently, steroid therapy of COVID-19 infections has been identified as another risk factor [41,42,43,44]. Molecular diagnostic approaches for the early and reliable diagnosis of systemic infections are presently under investigation [45].

*Lichtheimia* spp., among others, have been reported to be associated with species-dependent human pathogenic potential [46,47,48,49,50,51,52,53], including rhino–cerebral mucormycosis [54]. As is typical for mucormycoses, *Lichtheimia* spp. infections have been predominantly described for severely immunocompromised patients [55,56,57,58,59], premature newborns [60], severely burnt individuals [61,62,63], or post-traumatic medical conditions [64]. Co-infections with other molds have been recorded [65], as has a probable nosocomial transmission in an intensive care unit [66]. Additionally, of note, farmer’s lung disease has been associated with *Lichtheimia* spp. antigens [67,68,69,70].

Frequent sources of transmission of non-nosocomial Mucorales infections comprise, in descending order of frequency, contaminated air, traumatic inoculation of soil or foreign bodies, and contact with or the ingestion of contaminated plant material [71]. Accordingly, environmental exposure presents a risk for clinically relevant infections with Mucorales in susceptible individuals because of the wide occurrence of Mucorales in soil [72].

Here, we describe an environmental investigation undertaken in order to control the risk of infection by Mucorales and other molds for such an individual with predisposing risk factors.

## 2. Materials and Methods

### 2.1. Medical Background of the Environmental Investigation

Transient colonization with *Lichtheimia corymbifera* of the nostrils of a teenage female patient who required peritoneal dialysis due to an underlying medical condition, along with chronic nasal colonization of her mother with the same fungal pathogen, triggered an environmental investigation. There were no signs of hypersensitivity, such as the presentation of farmer’s lung disease, in either the patient or her mother.

The aim, by means of exposure prevention, was risk reduction for the girl, whose need for peritoneal dialysis signified a risk of progression of Mucorales colonization to invasive disease [6,37,38,39,40].

### 2.2. Environmental Investigation and Laboratory-Based Work-Up

Measurements of both airborne and surface-related mold spore loads at the living and working places of the family in a rural setting of northern Germany were conducted as part of the environmental investigation, comprising a screening of the family’s house and garden as well as the working places, a nearby pigsty, and a cow barn.

The techniques used were airborne spore collection using an Air Sampler RCS Plus device (Biotest Diagnostics, Dreieich, Germany), as well as the smear testing and plating of suspicious material on Sabouraud dextrose selective agar (Becton Dickinson, Heidelberg, Germany). The airborne spores were obtained with the Air Sampler RCS Plus device at a distance of 1.5 m from the floor. The assessed air volumes were 100 L in each case; the results of the airborne measurements were normalized to 1 m^3^. All Sabouraud dextrose agar plates were incubated for 2 days at 36 °C, and for a further 5 days at room temperature. The weather conditions were assessed using standard commercial thermometers, hygrometers, and barometers.

Cultural growth on Sabouraud dextrose agar (Becton Dickinson), as well as micromorphological differentiation of growing molds, was conducted in a microbiological diagnostic laboratory accredited according to DIN EN ISO 15189 and according to the locally established standard operating procedures. It was attempted to isolate microscopically identified Mucorales fungi and then to differentiate them by 18S rRNA gene sequencing, as described elsewhere [73].

## 3. Results

### 3.1. Descriptive Assessment of the House and the Garden of the Patient’s Family and of the Occupational Settings

**Figure 1:**
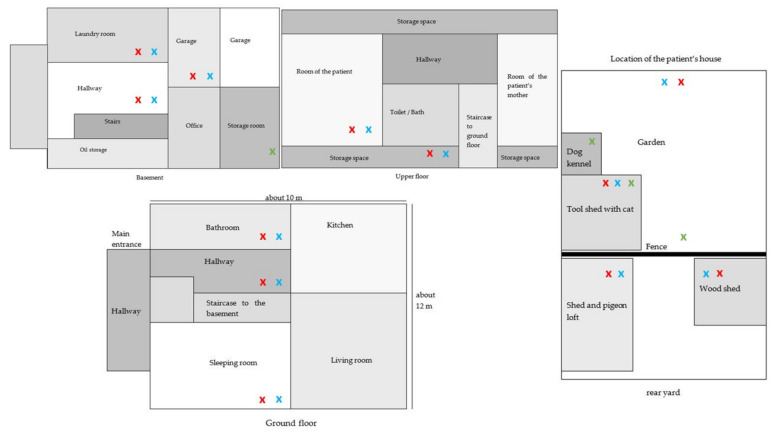
Sketch of the patient’s house and its surroundings. Sites of measurement of the weather conditions during the assessment are marked with blue “x” symbols, sites of airborne pathogen collections with red “x” symbols and points of smears or similar sample taking with green “x” symbols.

The patient’s home was a detached, three-story residential building. It was a prefabricated house with a full basement made of bricks, 28 years old, and was last renovated 18 years prior to the assessment. There was insulation of the exterior walls but no roof insulation. The basement comprised two garages, a laundry room, and an office room. On the ground floor there were six rooms, and on the upper floor, four rooms. Above the upper floor, a single-roomed attic could be reached by a ladder. Details are provided by the sketches in Figure 1.

The family was instructed to keep the windows and the doors closed for at least 12 h before the survey. The windows consisted of insulating glass to all sides. Potentially uninterrupted ventilation via an open fire place in the living room was noted, and a smell of oil and chemicals in each basement room and a musty smell and visible salt blooms in the storage room in the basement were detected. Of note, the basement and the attic were only occasionally used, so that these rooms were hardly ventilated.

The solid floors in each story were made of concrete, partly complemented with tiles. Linoleum laminate covered the floor in the patient’s room; there was carpeting in other rooms. The residence’s walls were partly covered with exposed plaster, partly with wallpaper. In the patient’s parents’ bedroom, the closets reached up to the ceiling and were positioned close to the walls, impeding ventilation in this room.

In the adjoining garden, there was a dog kennel, a shed housing young cats, a pigeon loft, and a woodshed. Pets (dog, cat, and pigeons) roamed in the immediate vicinity of the property. Organic waste was accumulated on a compost heap in the garden. Details of the garden and front yard are illustrated in the sketch in Figure 1. Recyclable waste was collected in the basement for up to 1 week at a time. The rooms could be heated by radiators; no humidifiers were detected. Fifteen potted indoor plants were distributed within the residence.

Moisture stains smaller than a postcard size were observed in the basement. One spot of visible mold growth was detected in the basement storage room, and a sample of the peeled wallpaper was taken with sterile tweezers from the moldy area. Otherwise, there was no visible fungal growth anywhere in the patient’s residence.

Further relevant features included a smoking habit of the patient’s father. Once a week, the house was cleaned by mopping and vacuuming. The floor of the patient’s room and the dialysis equipment were disinfected in addition to regular cleaning.

The occupational settings assessed comprised a nearby pigsty and a cow barn; their dates of construction were unknown. The cow barn was last renovated one year prior to the assessment. The windows and doors of the pigsty and cow barn were regularly closed. Within the cow barn, a moldy area smaller than DIN A4 size was visible on the ceiling of the milking barn section.

**Figure 2:**
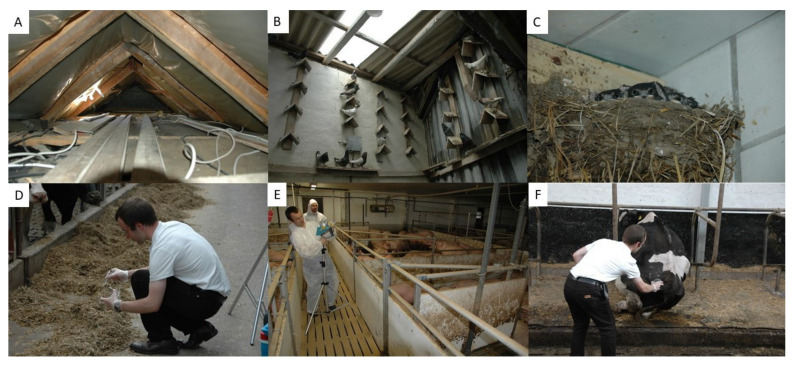
Illustration of the sampling sites and sample acquisition. (**A**) Attic. (**B**) Pigeon loft. (**C**) Bird nest within the barn. (**D**) Sample acquisition of straw in the barn. (**E**) Airborne spore collection in the pigsty. (**F**) Direct assessment of surfaces with agar plates in the cow shed.

Sampling sites and sampling strategies are shown in Figure 2.

### 3.2. Climatic Conditions at the Time of the Assessment

The assessment was performed on a cloudy, thundery summer day. A thunderstorm with considerable air movement was recorded towards the end of the measurements in the pigsty and the cow barn. Increased air pressure, a temperature drop of about 5 °C, and an increase in relative humidity by 10% to 20% were recorded. Details are provided in Table A1 of Appendix A.

### 3.3. Diagnostic Results of Cultural Growth

In the airborne pathogen collection, it was necessary to deviate from the standard distance of 1.5 m of the Air Sampler RCS Plus device from the floor in two instances: (a) storage room (dormer), device standing directly on the floor; (b) wood shed, device standing directly on the wood. Details of the results of the screening for airborne pathogens are provided in Table 1.

In addition to the outside air measurements, further samples were collected, and smear tests were performed. Details of the results are provided in Table 2.

In summary, 18S rRNA gene sequencing for species identification was attempted from the Mucorales isolates from the pigeon loft, from the upper floor storage room, and from the straw from the cow barn, but contamination-associated poor sequence quality allowed species identification of *Rhizopus arrhizus* only from the pigeon loft samples. *Lichtheimia corymbifera*, which was isolated from the human samples, could not be identified in the environmental specimens. Microscopical assessments of conidia did not lead to conclusive results.

## 4. Discussion

### 4.1. Summary and Interpretation of the Results of the Environmental Screening

Given that the spore counts per cubic meter of indoor air were identical to or lower than those of outdoor air, the indoor deposits of mold spores in the family house as well as in the pigsty and in the renovated part of the cow barn were most probably due to contamination from outdoor air. In support of this, in the pigsty, good correlation was found between agents from indoor and outdoor air, even at the species level. Clearly, there was no qualitative or quantitative evidence for the indoor growth of any mold species.

The relatively low number of spores in the air of the attic most likely reflected its infrequent contact with both the outdoor and indoor air of the occupied spaces. On the other hand, the tenfold higher spore load of the indoor air in the old cow barn as compared to the outdoor air strongly indicated the autochthonous growth of molds. The standard threshold for this assumption is an indoor air spore count exceeding the outdoor air spore count by a factor of two. Moreover, *Aspergillus* spp. and Mucorales were only detected in the indoor air of the old cow barn.

Additionally, the indoor air spore count in the pigeon loft exceeded the outdoor reference by a factor of >2.5. Although the Mucorales detected there turned out to be *Rhizopus arrhizus*, such results imply relevance as a potential source of additional Mucorales exposure.

For safety reasons, it was further recommended that the storage space on the first floor contaminated with Mucorales spores should not be used as storage for peritoneal dialysis consumables.

The patient’s mother frequently spent time in the cow barn area due to her occupation; this occupational behavior was considered as the most likely source of the chronic colonization of her nostrils with Mucorales. The source of the colonization of her daughter’s nasal cavity, however, could not be determined from the investigation.

Only a few fungal spores could be grown from the damp wall areas with peeling wallpaper and wall paint that were found in the storage room of the cellar. Accordingly, those observations were interpreted as remnants of longer historic periods of high humidity, due either to condensation or to leakage. Additionally, the numerous plants as well as the woodpiles in front of the house seemed to be of little relevance as sources or reservoirs, considering the low spore counts measured there.

The investigations had several deficiencies. First, because of the high workload involved in even one investigation, and because the patient’s family also declined further investigations in their home, the sampling procedure was performed only once. This is adequate only to give a snapshot of a potentially dynamic situation. Repeated examinations are desirable, but most often are not performed in real-life settings.

Second, the failure of discrimination of several of the grown Mucorales at species level by the Sanger sequencing approach used is an admitted limitation of the assessment. As observed in a previous methodological study [73], minor contamination is sufficient to cause non-interpretable Sanger sequencing results, and isolation attempts on Sabouraud dextrose agar failed to ensure contamination-free pure cultures in several instances. Beyond the contamination issue, panfungal PCRs targeting ITS regions have been reported to be more reliable for the sequence-based discrimination of Mucorales [74] than the 18S rRNA gene-based approach that was chosen. As reported elsewhere, specialized agar enriched with antifungal drugs may be applied to facilitate isolation attempts from environmental samples [72]; unfortunately, those approaches were not used for this assessment. Nor was a *Lichtheimia* spp.-specific real-time PCR [75,76,77] available, offering evaluated specificity in line with the requirements for diagnostic purposes in an accredited medical laboratory in Germany.

Third, initial growth temperatures lower than 36 °C would have facilitated the growth of Mucorales other than thermotolerant *Lichtheimia* spp. [78,79]. Thus, the diagnostic yield could have been higher than under the chosen conditions. However, even the sample growth of environmental fungi that was achieved made the identification of specific mold species challenging.

Despite the impossibility of identifying the environmental source of the *Lichtheimia* isolates, the sampling procedure distinguished several hot spots for mold spores in the immediate as well as in the more distant environment of the juvenile dialysis patient. In most cases, a higher spore count was not due to the local growth of fungi, but was most probably caused by the repeated trapping and/or sedimentation of spores from the outdoor air. The identification of such hot spots is important because molds, generally, have been reported to cause systemic infections in patients undergoing peritoneal dialysis [1,2,3,4,5,6]. With that knowledge, the family could be instructed on techniques to decrease the indoor spore count. Furthermore, the patient was counselled to at least avoid such hot spots in her household environment.

Generally, the sampling of environmental material is a process that can only be partially anticipated in standard operating procedures (SOPs), especially when a household and workplace situation is as diverse as in the present case. Therefore, when addressing the overall benefit of such inspections as described here, these reports help laboratories that do not perform such investigations on a daily basis to improve their SOPs and to prepare the sampling staff for potentially unexpected situations.

### 4.2. Recommendations for the Patient and Her Family

In response to the environmental investigation, a number of procedures were recommended to the patient’s family in order to reduce or avoid contact with reservoirs of spores, and thus to minimize the risk for the patient herself. First, intensified cleaning in combination with the intermediate thorough aeration of rooms with high spore loads was suggested to the family members. Second, the storage of dialysis consumables in the first floor storage area contaminated with Mucorales should be eliminated. Third, the need to keep a pigeon loft, where high concentrations of potentially etiologically relevant molds were detected, should be reviewed with the consideration of alternatives, such as buying pigeon meat from the market. Finally, the patient herself was recommended to stay away from the pigeon loft and also to avoid the cow barn area if possible, in order to decrease her exposure to high mold spore concentrations.

Due to lack of follow-up consultations with the patient or her relatives, no repeat analyses were performed. Thus, no conclusions could be drawn on the dynamics of the spore contamination or even colonization of the patient’s environment with molds in general or Mucorales in particular, or on the potentially beneficial consequences of following the recommendations.

### 4.3. Reasons for the Recommendations

Due to the medical history of chronic renal failure requiring peritoneal dialysis, the patient for whom the environmental investigation was performed was at risk of acquiring clinically apparent invasive mycosis due to the colonizing Mucorales [6,37,38,39,40], but also due to other molds. The chronic colonization of the mother’s nostrils as well as the transient colonization of the patient’s nostrils suggested exposure to an environmental source. Complete removal or—if impossible—at least avoidance would have reduced the risk. Although the colonizing *Lichtheimia corymbifera* was only detected in the mother’s nostrils during family screening, not specifically in the environment, increased concentrations of other molds—including species of the order Mucorales—were discovered that could pose an independent risk to the patient’s health, justifying the above recommendations, including the restriction of movement of the patient at a few specific sites of her household.

## 5. Conclusions

This report illustrates an environmental investigation to facilitate risk-adapted exposure prevention for a patient at risk of acquiring invasive mycosis caused by molds. The aim of such work-intensive procedures is the specific identification of risky sites, and thus the formulation of targeted recommendations that restrict the patient’s personal freedom as little as possible. Future investigations should explore soil samples as a typical habitat [72] for increasingly sensitive Mucorales detection. Repeated reports on Mucorales infections from environmental sources [71] have suggested the advantage of such approaches. Additionally, the potential risk resulting from the presence of other molds such as *A. fumigatus* [1,2,3,4,5,6] could be simultaneously assessed and addressed by the recommendations.

## Figures and Tables

**Table 1:** Mold detections from the airborne pathogen samples. Due to the nasal colonization of the index patient, Mucorales are indicated in **bold** type.

Measuring Point	Spore Quantity (cfu/1 m^3^)	Detected Mold Species	Comments
Residence, ground floor, hallway	120	*Altenaria* spp., *Mycelia sterilia*	-
Residence, ground floor, bathroom	10	*Aspergillus nidulans*	-
Residence, ground floor, sleeping room	160	*Penicillium* spp., *Mycelia sterilia*	-
Residence, upper floor, the patient’s room	30	*Penicillium* spp., *Mycelia sterilia*	-
Residence, upper floor, storage room (oriel)	10	**Mucorales**	Species differentiation by 18S rRNA gene analysis failed
Residence, attic	60	*Scopulariopsis brevicaulis, Mycelia sterilia*	-
Residence, basement, hallway	140	*Altenaria* spp., *Mycelia sterilia*	-
Residence, basement, laundry room	20	*Aspergillus fumigatus*	-
Residence, basement, garage	100	*Penicillium* spp., *Mycelia sterilia*	-
Residence, outside air measurement (garden)	200	*Penicillium* spp.	-
Garden, pigeon loft	520	*Aspergillus flavus, Rhizopus arrhizus*	Differentiation of the *Rhizopus* spp. by 18S rRNA gene sequencing
Garden, shed with a cat	210	*Aspergillus flavus, Mycelia sterilia*	-
Garden, woodshed	10	*Mycelia sterilia*	-
Pigsty, anteroom	30	*Aspergillus fumigatus*	-
Pigsty, pigpen	20	*Aspergillus fumigatus*	-
Pigsty, silage room	20	*Aspergillus fumigatus*	-
Pigsty, outside air measurement	950	*Aspergillus fumigatus, Mycelia sterilia*	-
Cow barn—inside	70	*Aspergillus fumigatus, Aspergillus flavus, Aspergillus* spp.	-
Old cow barn—inside	1030	*Aspergillus fumigatus, Aspergillus niger*, **Mucorales**, *Aspergillus* spp.	Species differentiation of the Mucorales by 18S rRNA gene analysis failed
Cow barn—milking barn	50	*Aspergillus fumigatus, Penicillium* spp.	-
Cow barn, outside air measurement	120	*Penicillium* spp., *Mycelia sterilia*	-

cfu, colony-forming unit. spp., species (indicating that differentiation beyond the genus level failed or was not attempted).

**Table 2:** Mold detections from smear tests. Due to the nasal colonization of the index patient, Mucorales are indicated in **bold** type.

Sampling Site	cfu/Specimen	Detected Mold Species	Comments
Residence, basement, wallpaper	27	*Aspergillus fumigatus, Aspergillus flavus, Penicillium* spp.	-
Garden, smear test from the cat	14	*Aspergillus fumigatus, Aspergillus flavus, Aspergillus* spp.	-
Garden, smear test from the dog	1	*Mycelia sterilia*	-
Garden, collected straw	28	**Mucorales**, *Candida rucosa*	Species differentiation of the Mucorales by 18S rRNA gene analysis failed
Cow barn, ceiling of the milking barn	37	*Aspergillus fumigatus*, **Mucorales**, *Aspergillus* spp.	
Cow barn, smear test from a cow	146	*Candida krusei, Aspergillus fumigatus*, **Mucorales**	
Cow barn, smear test from another cow	42	*Saccharomyces cerevisiae, Aspergillus fumigatus,* **Mucorales**	

cfu, colony-forming unit. spp., species (indicating that differentiation beyond the genus level failed or was not attempted).

## Data Availability

All relevant data are provided in the manuscript.

## Appendix A

**Table A1:** Measurement of the weather conditions during the assessment.

Measuring Point	Air Pressure (kPa)	Temperature (°C)	Relative Humidity (%)
Residence; ground floor, hallway	999.3 hPa	23.5 °C	54.2%
Residence, ground floor, bathroom	999.3 hPa	23.8 °C	49.7%
Residence, ground floor, sleeping room	999.4 hPa	23.5 °C	42.8%
Residence, upper floor, the patient’s room	999.1 hPa	24.4 °C	49.8%
Residence, upper floor, storage room (oriel)	998.9 hPa	24.8 °C	49.6%
Residence, attic	998.9 hPa	25.9 °C	40.4%
Residence, basement, hallway	999.5 hPa	24.0 °C	53.1%
Residence, basement, laundry room	999.5 hPa	25.6 °C	54.3%
Residence, basement, garage	999.8 hPa	24.7 °C	50.0%
Residence, outside air measurement (garden)	1000.1 hPa	22.2 °C	45.8%
Garden, pigeon loft	1000.2 hPa	21.9 °C	47.3%
Garden, shed with a cat	1000.2 hPa	22.2 °C	50.8%
Garden, woodshed	1000.0 hPa	20.1 °C	55.6%
Pigsty, anteroom	1002.0 hPa	26.1 °C	47.4%
Pigsty, pigpen	1001.9 hPa	25.9 °C	46.1%
Pigsty, silage room	1002.2 hPa	26.6 °C	49.9%
Pigsty, outside air measurement	1002.9 hPa	19.8 °C	49.5%
Cow shed—inside	1002.4 hPa	20.3 °C	63.0%
Old cow shed—inside	1002.4 hPa	20.3 °C	63.0%
Cow shed—milking barn	1001.5 hPa	18.1 °C	77.1%
Cow shed—outside air measurement	1001.5 hPa	18.4 °C	71.2%
